# Host adaption to the bacteriophage carrier state of *Campylobacter jejuni*

**DOI:** 10.1016/j.resmic.2015.05.003

**Published:** 2015

**Authors:** Kelly J. Brathwaite, Patcharin Siringan, Phillippa L. Connerton, Ian F. Connerton

**Affiliations:** Division of Food Sciences, School of Biosciences, University of Nottingham, Sutton Bonington Campus, Loughborough, Leicestershire LE12 5RD, United Kingdom

**Keywords:** Campylobacter, Bacteriophage, Carrier state life cycle, RNA-seq

## Abstract

The carrier state of the foodborne pathogen *Campylobacter jejuni* represents an alternative life cycle whereby virulent bacteriophages can persist in association with host bacteria without commitment to lysogeny. Host bacteria exhibit significant phenotypic changes that improve their ability to survive extra-intestinal environments, but exhibit growth-phase-dependent impairment in motility. We demonstrate that early exponential phase cultures become synchronised with respect to the non-motile phenotype, which corresponds with a reduction in their ability to adhere to and invade intestinal epithelial cells. Comparative transcriptome analyses (RNA-seq) identify changes in gene expression that account for the observed phenotypes: downregulation of stress response genes *hrcA*, *hspR* and per and downregulation of the major flagellin *flaA* with the chemotactic response signalling genes *cheV*, *cheA* and *cheW*. These changes present mechanisms by which the host and bacteriophage can remain associated without lysis, and the cultures survive extra-intestinal transit. These data provide a basis for understanding a critical link in the ecology of the *Campylobacter* bacteriophage.

## 1 Introduction

The bacterial pathogen *Campylobacter jejuni* is a common cause of human diarrhoeal disease worldwide. Infection can arise from food and water-borne sources, but is notably associated with the consumption of contaminated poultry products [1]. The intestines of poultry are often colonised by campylobacters without compromising the health of the birds, but their presence represents a foodborne hazard to humans when transferred to poultry meat during processing. Bacteriophages have the potential to control bacterial pathogens, and the application of bacteriophages that predate campylobacters can reduce the intestinal carriage of poultry [2,3] and the contamination of poultry meat [4]. *Campylobacter*-specific bacteriophages can be recovered from the intestines of poultry where their host bacteria proliferate [5], and readily infect and replicate within campylobacters in the laboratory [6]. Under these circumstances the bacteriophages are exclusively virulent in that they propagate by infection and lysis of host bacteria. Consistent with a virulent life style, these bacteriophages have contractile tails and icosahedral head morphologies that would place them in the family *Myoviridae* as part of the T4-like phage superfamily [5]. More recently, a new subfamily, the *Eucampyvirinae*, has been proposed that consists of two genera: “Cp220likevirus” and ‘‘Cp8unalikevirus”, which characteristically possess genome sizes in the range of 130–140 and 170–190 kb respectively [7].

Treatments of *Campylobacter* biofilms with Cp8unalikevirus bacteriophages result in a reduction of the viable bacteria and dispersal of the matrix [8]. Within mature biofilms, bacterial growth is severely restricted, and it is from these restrictive conditions that we have reported the recovery of campylobacters that remain bacteriophage-associated in a relationship that has been referred to as the carrier state life cycle (CSLC) [9]. The CSLC has been observed with strictly lytic bacteriophages infecting various bacterial genera [10–17], and describes mixtures of bacteria and bacteriophages that persist in a more or less stable equilibrium [18]. In the *C. jejuni* CSLC cultures, the phage titres remained equivalent to the numbers of viable bacteria following repeated subculture, implying that phage replication was continuing within a subpopulation of sensitive cells, whilst the remaining bacteria were capable of evading phage infection despite their close proximity. The recovery of infectious bacteriophages following treatments of CSLC cultures with either chloroform or bacteriophage-neutralising antibodies established that the phage particles are pre-assembled and intimately associated with the bacteria in that they are sheltered from the antibody [9]. However, the association does not prevent the release of bacteriophages, which allows for free phage particles to explore the environment for new host bacteria. *C. jejuni* CSLC cultures could not efficiently colonise chickens, but when administered to *Campylobacter* pre-colonised chickens, the CSLC phage readily replicated to bring about a reduction in the intestinal counts of the resident population [9]. The dissemination of free phage particles, whilst maintaining host association of a subpopulation phage to ensure against the low probability of encountering permissive hosts upon entering extra-intestinal environments, constitutes a hedge betting strategy that would ensure survival of the phage. CSCL bacteria also have notable phenotypic changes compared to wild type bacteria. These include improved aerotolerance under nutrient-limited conditions, which would confer a survival advantage in extra-intestinal environments, and a lack of motility, which would account for their inability to colonise chickens [9].

This study aims to investigate changes in gene expression and regulation associated with *C. jejuni* CSLC host phenotypes and to understand how the hedge betting strategy of the bacteriophage is implemented.

## 2 Materials and methods

### 2.1 Campylobacter *strains and bacteriophages*

*C. jejuni* PT14 [19,20] was routinely grown on horse blood agar (BA) at 42 °C under microaerobic conditions for 18 h as previously described [2]. *Campylobacter* cultures were resuspended in Mueller-Hinton (MH) broth (CM0337; Oxoid, Basingstoke, UK) using a sterile swab to use as inoculums to initiate broth cultures. Microaerophilic conditions were maintained using anaerobic jars employing gas replacement (85% N_2_, 5% O_2_ and 10% H_2_).

The *Eucampyvirinae* Cp8unalikevirus bacteriophages CP8 and CP30A [8] were isolated in the UK from poultry excreta [2]. Cp220like virus bacteriophage CP220 [20,21] was isolated from poultry meat in the UK. These phages were propagated on the bacterial hosts to be embedded in soft agar overlay using NZCYM agar as previously described [6,22].

### 2.2 Growth characteristics of *C. jejuni* CSLC strains

To determine the primary characteristics C. *jejuni* PT14 and CSLC isolates were inoculated into 50 ml of sterile MH broth to final optical density (A_600_) 0.01–0.02 (approximately 10^5^ CFU/ml). For RNA extractions, the procedure was scaled-up to 500 ml MH broth cultures in 2 l flasks. Samples were incubated shaking at 42 °C under microaerobic conditions over 24 h. Aliquots of 100 μl were removed every 2 h and immediately tenfold serially diluted using maximal recovery diluent (MRD; Oxoid). Campylobacters were enumerated as described previously [8]. Briefly, serial tenfold dilutions were made in MRD (CM0733; Oxoid) and enumerated in triplicate on mCCDA (CM0739, Oxoid) agar with additional agar (L13; Oxoid) to a total of 2% (w/v) added to reduce swarming. Plates were incubated under microaerobic conditions at 42 °C for 48 h before typical *Campylobacter* colonies were counted. Bacteriophages were enumerated using the soft agar overlay method as previously described [22]. Briefly, serial tenfold dilutions of phage suspensions were applied as 10 μl droplets in triplicate to the surface of prepared host bacterial lawns and allowed to dry. Plates were then incubated under microaerobic conditions at 42 °C for 48 h before the plaques were counted.

### 2.3 Carrier state and motility tests

The carrier state phenotype was defined by plaque formation following microaerobic incubation of a soft agar overlay containing the test isolate alone using the method described above. Motility was assessed by inoculation of 0.4% MH agar followed by incubation for 24 h under microaerobic conditions. Motility was assessed as a function of the radius of the motility halo with a strain being defined as motile if the halo radius exceeded 20 mm.

### 2.4 Cell culture, adherence and invasion

HCA-7 colonic epithelial cells [23] were grown as monolayer cultures in 24-well plates in Dulbecco's Modified Eagle's Medium (D-MEM) supplemented with foetal calf serum (FCS) at 10% (v/v) (Invitrogen Ltd) at 37 °C in 5% (vol/vol) CO_2_ [24]. Cell viability was monitored by performing microscopic examination of 0.4% Trypan-Blue-stained cells. Duplicate cell monolayers at 70% confluence were covered with bacterial cells at an approximate multiplicity of infection of 100 in D-MEM and incubated at 37 °C for 3 h in 5% (vol/vol) CO_2_. One monolayer plate was then washed 3 times with sterile phosphate-buffered saline (PBS), monolayers were lysed from one of the plates by adding 0.1% (vol/vol) Triton X-100 and viable bacterial counts enumerated on mCCDA plates recorded as the total adherent and invaded cell numbers. The monolayers of the second plate were similarly washed with PBS and 1 ml of fresh D-MEM supplemented with 250 μg gentamicin ml^−1^ and incubated for a further 2 h to kill all extracellular bacteria. The monolayers were then washed three times with PBS and lysed with 0.1% (vol/vol) Triton X-100 in PBS to release the internalized bacteria, which were enumerated on CCDA plates. The adherent bacterial count was derived by subtracting the number of internalized bacteria from the total and expressed as the percentage of the inoculum. The invasion efficiency was expressed as the percentage of the inoculum that survived gentamicin treatment. The experiments were recorded as the means of triplicate experiments.

### 2.5 RNA isolation and purification

Total RNAs were extracted from three independent early-log phase cultures of PT14, PT14CP8CS and PT14CP30ACS using the TRIzol^®^ Max™ bacterial isolation kit with Max™ bacterial enhancement reagent (Invitrogen, Paisley, UK) according to the manufacturer's instructions and the quality controlled as previously described [25]. The RNAs were ethanol-precipitated before purification using the RNeasy^®^ mini-kit of Qiagen (Crawley, UK) according to the manufacturer's instructions. On-column DNase treatment was included during purification using the RNase-free DNase set (Qiagen). The purified total RNAs were assessed for quality using an Agilent 2100 Bioanalyzer (Agilent Technologies Inc., South Queensferry, UK) with Prokaryote Total RNA Nano series II software, Version 2.3. RNA samples with an RNA integrity number (RIN) of 7.0 and above were selected for cDNA library preparation. Total RNA yields were determined using an ND-1000 spectrophotometer (NanoDrop Technologies).

### 2.6 Library construction and transcriptome analysis

Total RNAs were depleted of rRNA using Ribo-Zero^TM^ rRNA removal kit for Gram-negative bacteria (Epicentre Biotechnologies, Madison, WI, USA). The removal of 23S, 16S and 5S rRNAs were confirmed using a Bioanalyzer 2100 (Agilent). For all samples, 2 μg of rRNA-depleted RNA was then used to prepare cDNA libraries with the TruSeq™ RNA sample preparation kit (Illumina, San Diego, CA, USA) following the manufacturer's instructions. Each library was individually indexed with a unique identification adapter sequence before 15 cycles of PCR enrichment. The libraries were validated using the MultiNA analyzer (Shimadzu Corporation) and were normalised to approximately 10 nM. The indexed libraries were pooled and loaded onto a single lane of an Illumina HiSeq 2000 flow cell at a final concentration of 7 pM.

### 2.7 Filtering and mapping of sequence reads

The raw sequence reads were imported into the CLC Genomics Workbench 6.0 package where filtering removed low-quality reads and trimmed the TruSeq™ adapter indexes. A duplicate read removal plug-in was also downloaded into the Workbench in order to filter reads for comparative transcriptome analyses. The filtered reads were analysed using the RNA-seq feature of the Genomics Workbench. Ignoring non-specific matches, reads were mapped to the *C. jejuni* PT14 genome sequence (GenBank accession number CP003871), and either of bacteriophages CP8 (GenBank accession number KF148616) or CP30A (GenBank accession number JX569801). Quality control of the sequences derived from replicate cDNA libraries were analysed by principal component analysis available within CLC Genomics Workbench.

### 2.8 Transcriptome studies

The number of reads per kilobase of transcript per million mapped reads (RPKM; [26]) were used as the means of determining expression levels and normalizing read counts. Differential expression was determined within CLC Genomics Workbench using the RPKM expression values in conjunction with Baggerly's test statistics [27]. To control the error rate, p-values were calculated using the False Discovery Rate (FDR) procedure as described by Benjamini and Hochberg [28]. Genes with a fold change of 2 or more and a corrected p-value of <0.05 were considered to exhibit differential expression and represented using Circos diagrams [29].

### 2.9 Real time qRT-PCR

Total RNAs were converted to cDNA using the Omniscript cDNA synthesis kit (Qiagen) according to the manufacturer's protocol. Specific primers for qRT PCR were designed with lengths of 18–24 nucleotides and melting temperatures ranging between 57 and 59 °C. Aliquots of cDNA were used as the template for qRT PCR. An optical 46-well micro titre plate (Applied Biosystems) was used with a 20 μl reaction volume consisting of Power SYBR^®^ Green PCR master mix (Life Technologies), 50 nM gene specific primers and the template. An ABI Prism 7000 sequence detector (Applied Biosystems) was programmed for an initial set up of 30 s at 95 °C, followed by 40 cycles of 15 s at 95 °C and 1 min at 57°- 58 °C. SYBR Green detects double-stranded DNA. A melt curve was obtained from a first step starting from 50 to 95 °C to control specificities of quantitative PCR reaction for each primer pair. Cycle threshold (CT) values were determined using Step one software version 2.0 (Applied Biosystems). The comparative threshold cycle method was used to calculate change (*n*-fold) where samples were normalised to the internal control product of the *pks* gene, which showed no change in expression levels between RNAseq samples. Reactions were performed in triplicate. The fold changes were calculated using the 2ΔΔCt method. The primer pairs utilised in the qRT PCR experiments to determine the change in expression for selected genes are given in Supplementary Table 1.

## 3 Results

### 3.1 Growth characteristics of *C. jejuni* CSLC strains

The association of bacteria and bacteriophages in the *C. jejuni* CSLC is characterised by maintenance of equilibrium between the *Campylobacter* viable counts and the phage titre upon serial passage. However, it is evident from laboratory broth cultures that phenotypic variants arise that can be recovered at different stages of growth [9]. To enable meaningful comparison of the transcriptomes of CSLC cultures with wild type *C. jejuni* PT14 from which they were derived, we correlated the growth of CSLC cultures with the time-dependent appearance of motile bacteria and a reduction in the proportion of the population representing the carrier state. Early exponential growth rates between 2 and 4 h for wild type *C. jejuni* PT14 (0.417 h^−1^ ± 0.055) and the carrier state cultures carrying bacteriophages CP8 (PT14CP8CS; 0.441 h^−1^ ± 0.033) and CP30A (PT14CP30ACS; 0.422 h^−1^ ± 0.032) at 42 °C in Mueller-Hinton broth under microaerobic conditions were not significantly different. Phenotypic variation was examined at each time point by selecting discreet single colonies from the enumeration plates and independently testing for their motility and bacteriophage association upon subculture. All the bacteria recovered at 4 h were bacteriophage-associated and non-motile, exhibiting phenotypes consistent with the CSLC. Motile bacteria appeared at 6 h, followed by a rise in the phage titres, presumably because these bacteria can be infected by bacteriophage and lysed. For all subsequent experiments, the biological replicates represent broth cultures harvested in early-log phase before the appearance of phage-free motile bacteria.

### 3.2 CSLC cultures are impaired in adhesion and invasion of intestinal epithelial cells

**Fig. 1:**
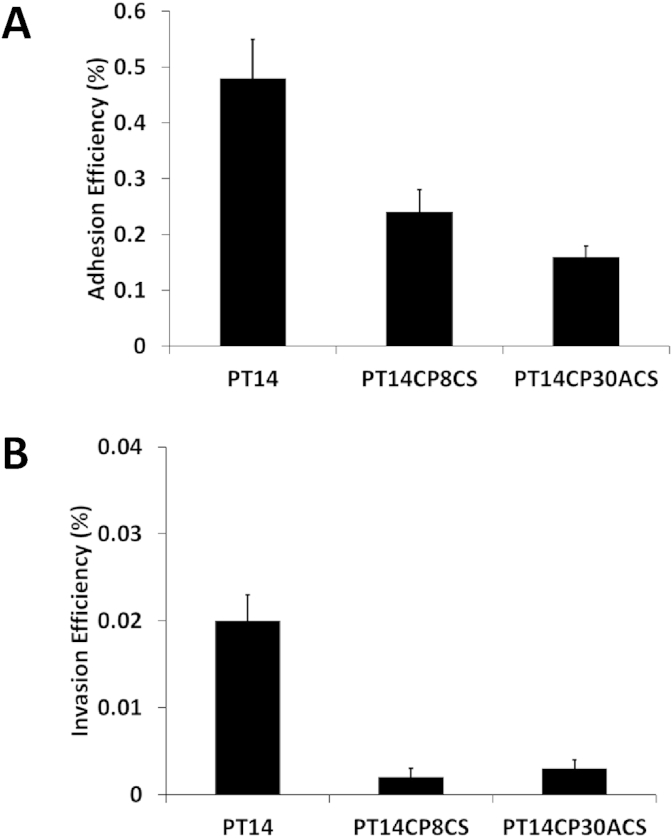
**Adhesion and invasion of colonic epithelial cells by CSLC cultures**. Adhesion (A) and invasion (B) of HCA-7 colonic epithelial cells for wild type PT14 and CSLC derivative *C. jejuni* strains. The adhesion efficiency represents the percentage of the inoculum remaining cell-associated less the internalized bacteria. The invasion efficiency represents the percentage of the inoculum that survived gentamicin treatment. The experiments are recorded as the means ± SD of triplicate experiments.

Flagellin mutants of *C. jejuni* are deficient in their ability to adhere to and invade cultured mammalian cells [30], and mutations that generally effect motility are accompanied by non-invasion phenotypes [31]. To determine if the non-motile nature of the CSLC cultures would also impact on their ability to adhere to and invade intestinal epithelial cells, a series of combined adherence and invasion assays were performed with wild type and CSLC cultures. Fig. 1 shows that CSLC cultures are significantly reduced in their abilities to adhere to and invade HCA-7 colonic epithelial cells compared to wild type *C. jejuni* PT14 (p < 0.01).

### 3.3 Transcriptome analysis of CSLC cultures

Using the annotated genome of *C. jejuni* PT14 as a basis for comparison; differential transcription was analysed between the PT14 CSLC strains and the parent strain by RNA-seq. Three replicate cultures for each strain were harvested at the early exponential phase of growth (4 h) incubated under microaerobic conditions at 42 °C in Mueller-Hinton broth as described above. The complete RNA-seq dataset is available in Supplementary Table 2 that represents between 7 and 16 million reads for each of three biological replicates for PT14, PT14CP8CS and PT14CP30ACS.

**Fig. 2:**
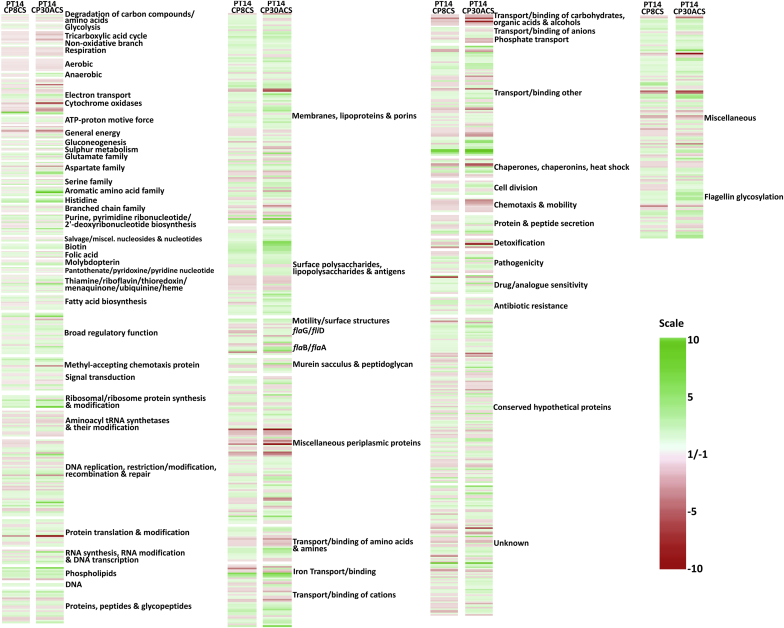
**Heat map showing genome-wide expression of the carrier state cultures compared to *C. jejuni* PT14**. Heat map comparing fold-changes in gene expression of the carrier state strains PT14CP8CS or PT14CP30ACS to the parent strain. The genes are first arranged according to their functional groupings and then according to chromosome position. The heat map colouring indicates increased expression in the carrier state cultures in green and decreased expression in red with respect to *C. jejuni* PT14. (For interpretation of the references to colour in this figure legend, the reader is referred to the web version of this article.)

**Fig. 3:**
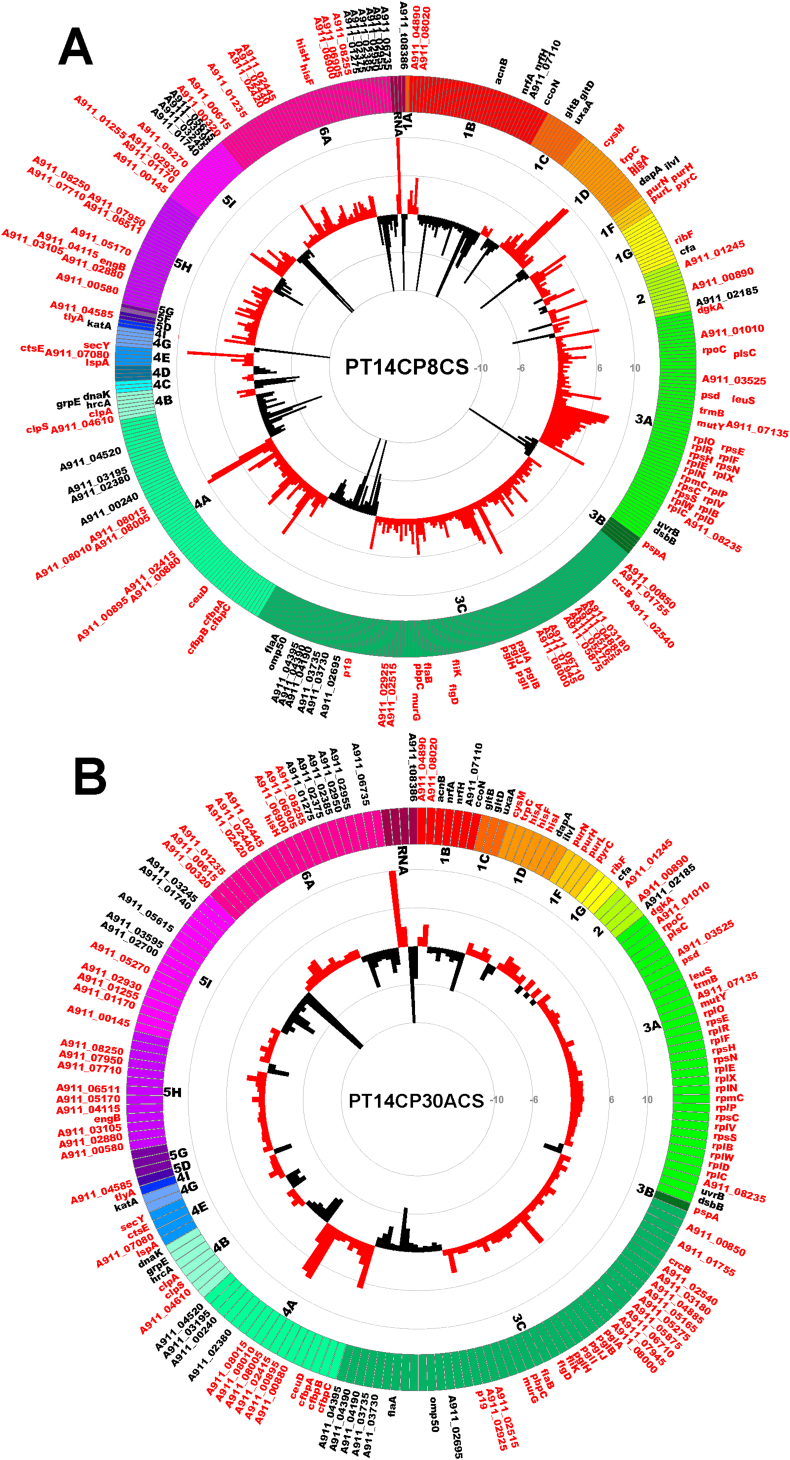
***C. jejuni* PT14 genes differentially expressed in the phage carrier state ordered by functional category**. Individual genes that were found to be greater than 2-fold differentially expressed in PT14CP8CS (A) or PT14CP30ACS (B) compared to non-infected bacteria are represented using Circos diagrams [29] with each gene represented and colour-coded according to functional class (http://www.nature.com/nature/journal/v403/n6770/suppinfo/403665a0.html) as follows: 1A) degradation; 1B) energy metabolism; 1C) central intermediary metabolism; 1D) amino acid biosynthesis; 1E) polyamine synthesis; 1F) purines, pyrimidines, nucleosides and nucleotides; 1G) biosynthesis of cofactors, prosthetic groups and carriers; 2) broad regulatory functions; 3A) synthesis and modification of macromolecules; 3B) degradation of macromolecules; 3C) cell envelope; 4A) transport/binding proteins; 4B) chaperones; chaperonins, heat shock; 4C) cell division; 4D) chemotaxis and mobility; 4E) protein and peptide secretion; 4G) detoxification; 4I) pathogenicity; 5A) IS elements; 5D) drug/analogue sensitivity; 5G) antibiotic resistance; 5H) conserved hypothetical proteins; 5I) unknown; 6A) miscellaneous; RNA) tRNA and rRNA. Only genes that are differentially expressed in both PT14CP8CS and PT14CP30ACS are labelled by locus-tag on the outer circle in red type for increased expression or black type for decreased expression. Histograms in the central rings represent the fold-change with increased expression in the carrier state compared to PT14 represented by red bars and reduced expression represented by black bars. Scale indicated by grey circles with values greater than 10-fold truncated, so bars touching the inner or outer rings represent a fold change ≥ -10 or 10 respectively. (For interpretation of the references to colour in this figure legend, the reader is referred to the web version of this article.)

Differential transcription of the CSLC strain PT14CP8CS compared to PT14 identified 319 genes showing 2-fold or greater upregulation and 146 genes showing 2-fold or greater downregulation with p-values <0.05 calculated using False Discovery Rate correction [28]. A similar analysis with PT14CP30ACS identified 137 genes showing ≥2-fold upregulation and 61 genes showing ≥2-fold downregulation. Fig. 2 presents a heat map of the normalised expression ratios of the carrier state cultures over the *C. jejuni* PT14 from which they were derived. There are striking similarities in the transcriptional response of PT14 to the carrier states induced independently by bacteriophages CP8 and CP30A, which share 113 upregulated genes and 43 downregulated genes. Fig. 3 presents a summary of the genes showing significant differential regulation for the carrier state cultures similarly ordered according to their functional classifications. Overall transcription within the carrier state cultures harvested at early stationary phase are markedly similar, but there are a few exceptions, as highlighted in Fig. 2. These data were used to explore similarities with transcriptomic data reported in the literature for defined regulatory networks in C. *jejuni*. A search of a curated database created in Microsoft Access revealed four regulons, within which the constituent genes exhibited co-ordinate transcriptional differences that were accompanied by changes in the expression of the corresponding regulatory genes (Supplementary Table 3). The regulatory genes affected in the respective carrier state strains PT14CP8CS and PT14CP30ACS as compared to PT14 were: *hrcA* downregulated 6.1- and 3.2-fold (temperature-sensitive repressor that controls GroES and GroEL; [32]); *hspR* downregulated 1.7- and 1.9-fold (temperature dependent co-repressor with DnaK; [33]); *perR* downregulated 1.5- and 1.3-fold (metal responsive repressor of oxidative stress response; [34]) and *nssR* upregulated 1.7- and 1.6-fold (positive regulator of nitrosative stress response [35]).

Using the pathway tools compiled at KEGG (www.genome.jp/kegg/pathway) and Biocyc (Biocyc.org) for C. *jejuni* PT14, the impact of core transcriptional changes identified in carrier state strains on cellular metabolic functions was surveyed. Most notably, an increase in the structural components required for ribosome biosynthesis was observed, with significant coordinate increases in transcription of genes encoding ribosomal proteins (50S and 30S components) being accompanied by a general increase in nucleotide biosynthetic components. Preferences in the biosynthesis of amino acids were evident with downregulation of glutamate, aspartate, lysine and branched chain amino acid pathways, but upregulation of the histidine, tryptophan and cysteine pathways. Differences in cellular transport functions were evident with the general amino acid, peptide, proline and aspartate transporters being notably downregulated along with the C4-dicarboxylic acid and L-fucose transporters. In contrast iron enterochelin and ferric transport systems were upregulated. Components of the type II secretion pathway (*sec*E,F,Y and lipoprotein signal peptidase) were upregulated along with N-linked protein glycosylation functions. Despite expression of genes encoding the flagellar-associated type III secretion system, the gene encoding the major flagellin filament *fla*A was downregulated. The carrier state strains also showed an increase in expression of specific peptidoglycan synthesis genes (*mur*G and penicillin binding protein 2).

Clustered repetitively interspaced palindromic repeats (CRISPRs) and associated proteins represent a mechanism by which bacteria acquire resistance to evade bacteriophage and plasmid infection. The CRISPR array of *C. jejuni* PT14 is organised into four 36 bp direct repeats flanking three 30 bp spacer elements, which are adjacent to a *trans*-activated CRISPR RNA (tracrRNA). In common with CRISPR arrays reported for several *C. jejuni* strains [36,37], each direct repeat contains an independent promoter such that a single RNase III/tracrRNA-dependent processing event is required to create the mature crRNA that targets Cas9 endonuclease to invading DNAs. As reported previously, the *C. jejuni* PT14 CRISPR array is dominated by an unusual transcript arising within the second spacer to produce abundant spacer 3 crRNA that has 15-fold greater reads than spacer 1 and > 30-fold the adjacent structural gene encoding Cas2 (A911_07325) [38]. In the CSLC cultures, the dominance of this transcript from the CRISPR array is notably reduced, which coincides with a marked increase in antisense RNAs arising from within the adjacent *cas*2 gene. RNA libraries from CSLC cultures contain native crRNAs, but also new crRNAs, which suggests acquisition and expression of new spacers. As discovered within DNA sequences of the CRISPR arrays of multiply propagated CSLC cultures, the new crRNAs appear to be derived from the host bacterial chromosome and not the bacteriophage genome sequences with which the bacteria are associated [38]. The self-derived crRNAs are documented in Supplementary Table 4.

The RNA-seq reads from carrier state strains PT14CP8CS and PT14CP30ACS were also mapped to the corresponding annotated genome sequences of the bacteriophages CP8 and CP30A. Phage transcripts comprised 0.2% of the total unique reads, with the most abundant transcript corresponding to the major capsid proteins from CP8 and CP30. This level of transcription could be attributed to background bacteriophage replication within the culture, possibly as a result of a minor fraction of the bacterial population gaining motility ahead of the rest.

### 3.4 Validation of RNA-seq using quantitative real-time PCR (qRT-PCR)

In support of the RNA-seq data, the relative expression of 22 genes was confirmed by qRT-PCR. Transcription of the *pgk* gene (A911_06820), encoding the central glycolytic enzyme phosphoglycerate kinase, was selected as the control for qRT PCR studies, since it showed similar ΔCT values for *C. jejuni* PT14 and the carrier state cultures, and no significant differences in the RNA-seq reads between *C. jejuni* PT14, PT14CP8CS or PT14CP30ACS (see Supplementary Table S2). Supplementary Fig. 1 confirms trends observed in the transcriptomic study by comparing the log_10_ RNA-seq ratios of the carrier state cultures over *C. jejuni* PT14 and the corresponding qRT PCR ratios. Concordance between RNA-seq and the qRT-PCR experimental data sets was observed with R^2^ values of 0.99 for PT14CP8CS and 0.98 for PT14CP30ACS following linear regression.

**Fig. 4:**
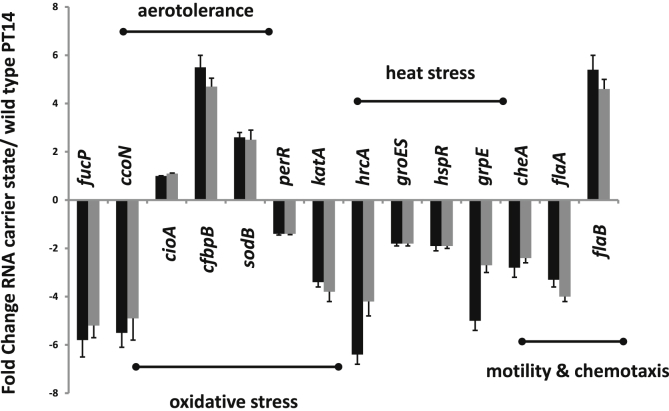
**qRT-PCR determination of the transcript levels of functional genes in the carrier state of *C. jejuni* PT14**. Carrier state transcript levels are expressed as fold-change over wild type *C. jejuni* PT14. The solid bars represent PT14CP8CS and the grey bars PT14CP30ACS. Transcription of the *pks* gene was used as an internal control for all assays. The genes are ordered according to the functions indicated above with the regulatory genes placed adjacent to their targets (*perR*/*katA*, *hrcA*/*groES*, *hspR*/*grpE*).

The transcript levels of key genes identified within the RNA-seq data that could affect phenotypic expression of carrier state cultures were further examined by qRT-PCR. Fig. 4 presents fold change in RNA levels of the carrier state genes of PT14CP8CS and PT14CP30ACS with respect to *C. jejuni* PT14. The genes are ordered according to the functional characteristics they encode and the regulatory networks to which they are ascribed. These data support observations made from RNA-seq highlighting similarities between the carrier state transcript levels of genes that affect selective nutrient uptake, responses to oxidative stress, heat stress, chemotaxis and motility. Notably, the differential expression of the *flaA* and *flaB* genes is also confirmed.

## 4 Discussion

We previously demonstrated that *Campylobacter* bacteriophages can enter into a complex relationship with their host. The association represents an alternative life cycle for virulent bacteriophages that effectively ensures that members of the phage population do not become dissociated from viable host bacteria. The continued propagation of host bacteria and bacteriophage is a hallmark of the carrier state life cycle that has been observed for several combinations of bacteria and virulent bacteriophages [10–17]. CSLCs likely represent an important link in the ecology of bacteriophages in transit between host-rich environments and those of low replication probability. The CSLC also represents a biotechnological opportunity to harvest bacteriophages in continuous production for diagnostic, biosanitisation and therapeutic applications [39]. However, the dynamics of bacteriophage infection and host survival give rise to phenotypic variance within CSLC cultures, which has made physiological investigation of these associations difficult. These processes were investigated to determine the optimum time to harvest for campylobacters from broth cultures that represent populations of CSLC bacteria synchronised with respect to their growth rate, phase of growth and phenotypes consistent with the CSLC (bacteriophage-associated non-motile bacteria). Using these criteria, we harvested campylobacters at 4 h and prepared RNAs to examine CSLC gene expression with reference to wild type *C. jejuni* PT14 by RNA-seq.

The status of the bacteriophage in these cultures is a key question, since our previous studies demonstrated that the phages were sheltered from neutralising antiserum treatment and that the cultures contain pre-assembled particles on the basis that they survive chloroform treatment that eliminates the host bacteria. However, chloroform treatment reduced the observed phage titre by >1 log_10_ PFU, suggesting that host-dependent replication occurs within the bacterial population [9]. RNA-seq analysis of early exponential PT14 CSLC cultures detected bacteriophage transcripts in all replicates. Phage transcripts comprised 0.2% of the total unique reads, values that were 40-fold down if proportional expression of the phage chromosomes could be achieved, given that their DNA content is 11.5-fold less than the bacterial chromosome. Nevertheless, the most abundant phage transcripts encoding the major capsid proteins of CP8 and CP30 (gp23 orthologues of T4 phage) are within the top 5% of all RPKM values recorded for carrier state cultures. The phage transcripts detected could represent the eclipse phase of a minor infected population or emanate from the largely transcriptionally inactive phage genome that is widely distributed in the population.

Campylobacters generally possess a minimal type II-C CRISPR-Cas system in which host RNase III functions to process crRNAs [36,37]. The genome of C. *jejuni* PT14 carries an intact CRISPR-Cas system that could function to inactivate bacteriophage genomes; however, the CRISPR array in PT14 does not produce crRNAs that target the carrier state bacteriophages, and the carrier state cultures derived from this strain show no evidence of the acquisition of phage DNAs in their CRISPR arrays. We recently reported that *Campylobacter* bacteriophages carry a conserved gene encoding a Cas4 orthologue that is otherwise absent in *C. jejuni* [38]. Cas4 functions as a 5ʹ-3ʹ exonuclease to create recombinogenic substrates for protospacer acquisition [40,41]. New cRNAs observed in these experiments represent bacterial host sequences rather than those of bacteriophage origin. This observation is consistent with the identification of host-derived spacers integrated into CRISPR arrays of multiply propagated CSLC cultures [38]. Self-spacer acquisition has been reported in other bacterial genera, but often leads to cell death as they become targets for host DNA cleavage by the CRISPR-Cas ribonucleoprotein complex [42,43]. However, the new crRNAs themselves have the potential to modify bacterial expression through the selection and expression of antisense RNAs. Among the crRNA antisense targets, the anti-terminator nusG is significant because it functions as a co-factor with Rho to suppress antisense transcription [44] and prevent bacteriophage gene expression [45]. Rather than eliminating the bacteriophage DNA using CRISPR-Cas, the pattern emerging from these studies is that of imposed tolerance through flexible RNA-based regulation. The transcription of several genes encoding RNase functions is differentially upregulated in the carrier state cultures; these include two RNase L-PSP family members, RNase BN, RNase H. and RNase III. RNase BN functions in *Escherichia coli* as an exo- and endoribonuclease with specific actions in the maturation of host and T4 phage tRNAs [46]. In this context, five phage-encoded tRNAs are expressed and processed in the carrier state, where the Met, Asn and Tyr tRNA genes will make significant contributions to the limited availability of these tRNAs from the host to achieve translation of host and phage proteins.

Motility is considered a critical factor in the ability of campylobacters to colonise the intestinal tract and cause pathogenesis [47]. CSLC campylobacters are non-motile with truncated flagella, which leave them unable to colonise chickens [9], and as demonstrated here, profoundly impaired in their ability to adhere and invade human intestinal epithelial cells. RNA-seq and subsequent qRT PCR analysis of the PT14 CSLC isolates revealed the gene encoding the major flagellin protein (FlaA) to be significantly downregulated in response to carriage of CP8 (2.3 or 3.3-fold respectively for RNA-seq and qRT PCR) or CP30A (4.1 or 4.0-fold respectively for RNA-seq and qRT PCR) as compared to wild type. Flagellar biosynthesis in *C. jejuni* is controlled the by the FlgSR two-component regulator, in which FlgS histidine kinase autophosphorylates to activate the FlgR response regulator. Phosphorylated FlgR works in conjunction with σ54-RNA polymerase holoenzyme to initiate transcription of genes encoding flagellar components and FlaB. FlgSR and σ54 are also required for full expression of σ28, which is required for expression of FlaA. In the carrier state cultures, σ54-dependent expression of the *flaB* gene appears to be functional, but σ28 mediated expression of the *fla*A gene is repressed, most likely by the anti-σ28 factor FlgM. The secretion of FlgM is required via a functional type-3 secretion apparatus to relieve repression of the *flaA* gene to enable production of the major flagellin FlaA and completion of functional flagella. In PT14CP30ACS, downregulation of genes associated with flagellar structure and function extended to the FlaG operon, featuring the flagella capping protein (FliD), and to the flagellar-secreted protein FspA2 that is co-regulated with FlaA and implicated in apoptosis of mammalian cells [48]. PT14 CSLC isolates are also likely impaired in their chemotactic response with the adjacent genes encoding the core signalling proteins CheV, CheA and CheW (A911_01365 - A911_01375), all showing significant downregulation (≥1.5-fold in PT14CP8CS and ≥2.2-fold in PT14CP30ACS). Three genes encoding chemoreceptors (methyl-accepting chemotaxis proteins; [49]) were also significantly downregulated in PT14 CSLC isolates: Tlp1 (A911_07255 responding to aspartate), Tlp6 (A911_02185 cytoplasmic transducing protein) and Tlp7 (A911_04600 responding to formate). The CSLC strains are severely compromised in their ability to colonise the intestines of host species, and yet they harbour and produce bacteriophages capable of infecting any new host-adapted campylobacters they encounter [9]. The competitive disadvantage imposed on the CSLC strains is further emphasised by 3- to 8-fold downregulation of all the L-fucose utilisation genes (A911_02350 - A911_02390) that are reported to confer a competitive advantage in the colonisation of pigs as a consequence of utilising L-fucose derived from intestinal mucin [50].

The association of the bacteriophage with the bacteria has the potential to generate greater phage diversity either via nucleotide modification, protein modification or cofactor binding such as metals [51]. The CSLC strains are significantly upregulated for a number of genes that could serve to modify phage components; these include five genes that could either methylate or glycosylate the phage DNA (SAM-dependent methyl transferases A911_02880, A911_06315 and A911_06906; adenine DNA methyltransferase A911_01010; A/G-specific glycosylase A911_07800), and at least 17 genes whose products could affect post-translational modification of protein (O-linked glycosylation A911_06480-06515; N-linked glycosylation A911_05425-05475; secreted serine protease A911_06330). It is also possible that DNA modification has a role in maintaining the phage in the carrier state if the phage chromosome is retained within the host, and discriminating it from any superinfecting phage. A range of restriction/modification systems arising from insertion or deletion have been observed in comparative genomic hybridization surveys of *Campylobacter* species, implying that different host bacteria have the capacity to encode the apparatus to support distinguishable mechanisms for the prevention of bacteriophage replication [52–54]. A high degree of diversity in the surface structures and metabolic profiles between *C. jejuni* strains also exists [55–57], and adding to this complexity, restriction/modification systems and surface structures in campylobacters have been observed to undergo phase variation to produce subpopulations in which these systems are ‘on’ or ‘off’ depending on a stochastic process [58]. Phase variation extends to capsular polysaccharide and flagella components with which bacteriophage may need to interact [59,60]. Phase variation may be considered as a population level defence against bacteriophage infection, but is interesting to consider that CSLC phages could perversely benefit from host-mediated phase variation to expand their structural and epigenetic diversity, and ultimately their host range.

We previously demonstrated that CSLC isolates are significantly more aerotolerant than the wild type from which they are derived under conditions of nutrient limitation, as would be experienced in extra-intestinal environments [9]. Subjecting carrier state cultures to these conditions is likely to modify gene expression compared to the transcriptome we observe from early-exponential phase cultures. These differences notwithstanding, our data provide evidence that carrier state cultures may be primed to withstand the physiological stresses imposed. *C. jejuni* PT14, like other *C. jejuni*, preferentially expresses a cbb3-type cytochrome c (*ccoNOQP*; A911_ 07165–07180) as a terminal oxidase (7-fold) over a cyanide-insensitive oxidase of the CioAB-type (*cioAB*; A911_00385 to A911_00390) that has a lower affinity for oxygen [61]. This preference is significantly diminished in the CSLC bacteria due to downregulation of the cbb3-type cytochrome c oxidase and either maintenance or upregulation of the CioAB-type. Under aerobic conditions, the relative increase in expression of a lower-affinity CioAB-type oxidase will resist oxygen saturation and damage. Downregulation of the higher oxygen affinity cbb3-type cytochrome c oxidase could also compromise the ability of carrier state bacteria to colonise low oxygen environments such as those encountered in the gut before finding a microaerobic niche against the gut mucosa, and will likely be a contributory factor in the observation that carrier state bacteria fail to colonise chickens [9].

The carrier state strains are downregulated for the autoregulatory repressor PerR [62], which controls expression of genes that mediate against oxidative stress. Like *perR*, the PerR-repressed genes encoding the major oxidative stress responsive proteins AhpC (alkyl hydroperoxide reductase) and KatA (catalase) are downregulated, but the oxidative stress-responsive proteins SodB (superoxide dismutase) and Tpx (thiol peroxidase) that are required to survive atmospheric oxygen [63,64] are significantly upregulated in the carrier state. PerR repression is iron-dependent and the CSLC cultures are notably upregulated for the genes required for iron acquisition. Since iron participates in the Fenton reaction to generate reactive oxygen species, it is critical that iron acquisition be coordinated with cellular activities that control and detoxify these species. The genes for iron uptake are regulated by Fur. The Fe^2+^-bound form of Fur or Holo-Fur acts as a repressor of the iron uptake systems, but in C. *jejuni*, the non-iron-bound form can also bind DNA and has been demonstrated to regulate diverse functions, including the transport of alternative divalent cations, flagellar and ribosome biosynthesis [65]. The differentially regulated genes observed in the carrier state have significant overlap with the Fur regulon, but not all iron-responsive genes are Fur-responsive [66]. The carrier state transcriptome implies that bacteria are exhibiting conditions of iron depletion most notably evident through upregulation of enterochelin (A911_06570 to A911_06585) and ferric (A911_00840 to A911_00845) transport systems. All components of flagella biosynthesis have been reported to be iron-activated [66], and are also notably downregulated in the carrier state. The nitrosative stress response activator *nssR* is upregulated in the CSLC cultures, which leads to strong transcription of genes encoding the oxygen binding globins Cgb (single-domain globin; A911_07630) and Ctb (truncated globin; A911_02270) [35], which may also act to confer greater protection against atmospheric oxygen. The globins and components of the CRISPR-Cas system contain iron centres critical for the structure and function of these proteins.

CSLC cultures are able to survive heat shock better than their parent bacteria under nutrient limitation and atmospheric oxygen. This was perhaps surprising given that transcriptomic data demonstrate that the PT14 carrier state cultures are repressed for the heat shock regulons controlled by HspR and HrcA. Mutation of *hsp*R relieves repression of the heat-shock-responsive operons *dnaK* (that contains *hrcA*), *cbpA* (that contains *hspR*) and *clpB*, but also result in significant downregulation of genes involved with motility [32,33]. Reduced expression of *hsp*R in the CSLC cultures is also accompanied by significant reduction in the expression of specific flagella genes that includes the major flagellin *fla*A, but not *fla*B. HspR functions by binding target promoters containing HAIR (HspR-associated inverted repeat) DNA sequences in complex with the chaperone DnaK, and is depressed by competition for DnaK binding when unfolded proteins accumulate following heat stress [67]. HrcA represses the *gro*E operon encoding chaperones GroES and GroEL, by binding at the DNA sequence element CIRCE (controlling inverted repeat of chaperone expression) within the promoter [32]. HrcA is heat-sensitive and will unfold upon heat shock, causing depression of the groE operon until sufficient GroEL/ES is available to fold HrcA. The heat shock repressors are dependent on chaperones they control, allowing for optimal response to stress. This fine tuning is likely modified in the CSLC because the phage genomes express chaperones, which include a functional substitute for co-chaperonin GroES [68]. Standing levels of phage-encoded chaperones would mean the CSLC cultures are primed to resist heat shock and will be able to respond further through derepression of HrcA as the chaperones are titred against unfolded protein substrates.

In summary, we defined culture conditions that enable RNAs to be extracted from synchronous populations of carrier state and wild type cultures, and for the first time allow comparative transcriptome analyses of bacteria in the carrier state. The carrier state is characterised by multiple regulatory changes in the host bacteria that would account for their ability to survive in extra-intestinal environments. These regulatory changes shape the ecology and evolution of campylobacters and their bacteriophages.

## Conflict of interest

The authors declare no conflict of interests.

## Appendix A Supplementary data

Table S1 Primer pairs utilised for qRT PCR to determine the change in expression for selected genes.

Fig. S1 **Correlation between RNA-seq and quantitative real-time PCR (qPCR)**. Log10^-^ transformed ratios of the mean normalised read counts obtained from RNA-seq for the carrier state cultures over *C. jejuni* PT14 are plotted against the corresponding ratios determined by qPCR. Locus-tag labels mark the data points showing differential gene expression. Regression lines and the corresponding r-squared values are included in the plots: A) PT14CP8CS; and B) PT14CP30AC.

Table S2 Comparative RNA-seq data for *C. jejuni* PT14, PT14CP8CS and PT14CP30ACS.

Table S3 Regulons that exhibit significant changes in PT14CP8CS and PT14CP30ACS.

Table S4 crRNA sequences of self-derived spacers expressed from PT14CP8CS or PT14CP30AC.
