# Supplementary material for: Host adaption to the bacteriophage carrier state of Campylobacter jejuni
Source: Res Microbiol. 2015 Jul-Aug;166(6):504–15. doi: 10.1016/j.resmic.2015.05.003 (PMC4534711; doi:10.1016/j.resmic.2015.05.003)
Supplement: Table S1 — Primer pairs utilised for qRT PCR to determine the change in expression for selected genes. [file mmc1.doc]

## Supplementary Table 1 Primer pairs utilised in qRT-PCR to determine the change in expression for selected genes.

| **Locus Tag**  **A911_** | **Target Gene** | **Forward Primer** | **Reverse Primer** | **Annealing Temperature (°C)** |
| --- | --- | --- | --- | --- |
| 00385 | *cio*A | TGGCGGTTGAAGGTATTATGG | AGCTCATTCCTACAGGATATTGC | 57 |
| 00815 | *sod*B | AAACTTCAAATGGGGGCGTAT | CACAGCCACAGCCTGTAC | 57 |
| 00840 | *cfbp*B | TCAAAGCCAAAAGGCGCTA | AGCGAGTGCAATTTATTCTTTAGAG | 57 |
| 01370 | *che*A | CGTTGCCATTATCACGAATGG | GTTAACTCACCACATGGAGGAT | 57 |
| 01555 | *per*R | GTTAAAAGCTACTCCGCAAAGAC | AATCCTCTATACCACCACATTTGG | 57 |
| 02375 | *-* | AAGCGAAGGCGGAATTCC | CATAGATTCCACCATGTTTGATAGC | 57 |
| 02380 | *fuc*P | TGGGGAGTAAGCTATGGACTT | GGCTATATAACCTGCGGGTAAG | 57 |
| 02485 | *clp*B | GCACCTAAGAGTCTTGAAACA | ACAATCGGCTAAAGCTTTGG | 57 |
| 02670 | *fli*M | GAAGCTAGTGGAGGAGAGATTG | CACTGTCTTCGCCTGTTTCT | 57 |
| 03465 | *rps*P | GTCGTGATGGTGGTTGGATAG | CTAAGTTTTGCACCAACGCTT | 57 |
| 03665 | *hrc*A | GAGGGTTTGATCACTCAGCTT | TCTTTTTCCCAAAAACTTTGCCA | 57 |
| 03670 | *grp*E | CAGCTGTTAATGTTGAATGCC | ATCTTTGATAAGAGCCACTCC | 57 |
| 05600 | *rho* | GGCGATATCGTTACAGGACAA | GTAAAGGCAGATAATTGATCGCTT | 57 |
| 05660 | *ded*A | TATAGCCACCCAAATAGCACTAC | CACGGGGAATTTTCAACCTTTAC | 57 |
| 05810 | *lux*S | ACCTATGGGTTGTCGCACG | TTTTGCTTTGATCGCTTACGC | 57 |
| 05920 | *gro*ES | AGCCTTTAATGGGTGAAGTAGTAG | CTGTTCCACCGTATTTAGCAAAC | 57 |
| 05970 | *hsp*R | CCAAGTAGAACCGATGGCAA | CAAGATTGATTCCCATGTCTCTTG | 57 |
| 06500 | *fla*B | ACTCATAGCATAAGAACCTGACTG | GTTAAAGCAGCAGAATCAACCA | 57 |
| 06505 | *fla*A | CCATGGCATAAGAGCCACTT | CAGCTGAGTCACAAATCCGT | 57 |
| 06730 | *kat*A | CAGGCGCAAAAGGACCTT | AGCTCCACTTCCCTTAGCAT | 57 |
| 06820 | *pgk* | AAGTCTAGCAAGACGCTTAGC | TAGACGCATAAGATCAGCTATTCC | 57 |
| 07175 | *cco*O | GACGAATAAGCTGTGAATGACAAG | GCAGGCATAGTTGAAGTTTTACC | 57 |
| 07185 | TCR | AATCTCTCTTGCCATATCCAGTC | ATCGGAGCTCAAAATGGAGATG | 57 |
| 08005 | - | GTAACAACATCACCGCACTTG | TCTCCTTGCACTGCTAAATAAGG | 57 |
