# Supplementary figures and images for: Host adaption to the bacteriophage carrier state of Campylobacter jejuni
Source: Res Microbiol. 2015 Jul-Aug;166(6):504–15. doi: 10.1016/j.resmic.2015.05.003 (PMC4534711; doi:10.1016/j.resmic.2015.05.003)

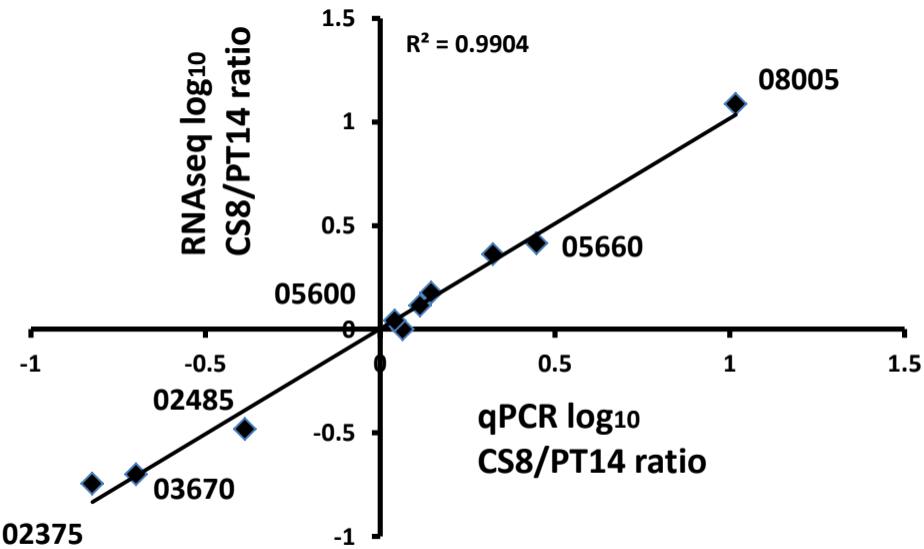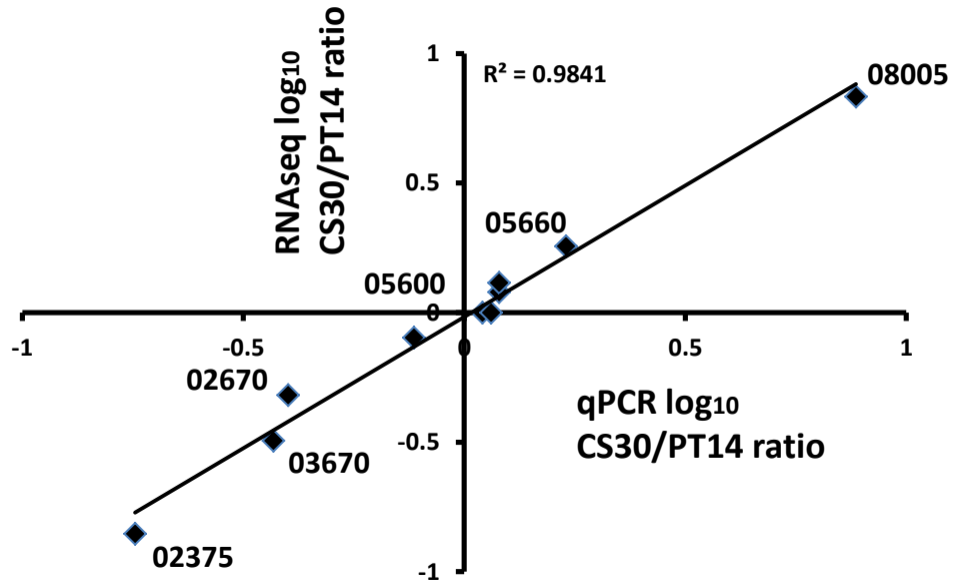

Supplement: Fig. S1 — Correlation between RNA-seq and quantitative real-time PCR (qPCR). Log10- transformed ratios of the mean normalised read counts obtained from RNA-seq for the carrier state cultures over C. jejuni PT14 are plotted against the corresponding ratios determined by qPCR. Locus-tag labels mark the data points showing differential gene expression. Regression lines and the corresponding r-squared values are included in the plots: A) PT14CP8CS; and B) PT14CP30AC. [file mmc2.pdf]
