# Supplementary material for: Host adaption to the bacteriophage carrier state of Campylobacter jejuni
Source: Res Microbiol. 2015 Jul-Aug;166(6):504–15. doi: 10.1016/j.resmic.2015.05.003 (PMC4534711; doi:10.1016/j.resmic.2015.05.003)
Supplement: Table S3 — Regulons that exhibit significant changes in PT14CP8CS and PT14CP30ACS. [file mmc4.docx]

# Table S2 Regulons that exhibit significant changes in PT14CP8CS and PT14CP30ACS

| **Regulon** | **Locus** | **PT14CS8CS fold change** | ***p*-value** | **PT14CS30ACS fold change** | ***p*-value** |
| --- | --- | --- | --- | --- | --- |

| HrcA | A911_00810 (Cj0168c) | 1.76 | 9.24E-09 | 1.57 | 3.82E-05 |
| --- | --- | --- | --- | --- | --- |
|  | A911_05920 (groES) | -1.70 | 3.88E-17 | -1.66 | 3.07E-13 |
|  | A911_05925 (groEL) | -1.98 | 0 | -1.90 | 0 |
| HspR | A911_00215 (Cj0044c) | 2.90 | 0 | -3.89 | 2.44E-24 |
|  | A911_02210 (thiC) | -1.51 | 3.45E-19 | -1.23 | 3.97E-05 |
|  | A911_03345 (flgH) | 1.75 | 6.42E-04 | 2.06 | 0.144791124^*^ |
|  | A911_03400 (flgG2) | 1.35 | 8.16E-03 | 1.64 | 0.180539551 |
|  | A911_04285 (flgL) | 2.01 | 6.14E-10 | 1.42 | 0.158689108 |
|  | A911_06030 (Cj1242) | 1.90 | 8.44E-03 | 1.92 | 3.07E-03 |
|  | A911_06500 (flaB) | 5.47 | 0 | 3.06 | 0 |
|  | A911_06505 (flaA) | -2.33 | 6.97E-32 | -4.07 | 1.94E-67 |
|  | A911_06555 (Cj1349c) | 1.84 | 3.69E-14 | 1.62 | 1.08E-08 |
|  | A911_06625 (Cj1364c) | 1.43 | 5.26E-15 | 1.31 | 1.33E-05 |
|  | A911_06970 (Cj1450) | 1.58 | 0 | -1.19 | 2.21E-04 |
|  | A911_07045 (flgI) | 1.34 | 8.09E-05 | 1.83 | 3.58E-02 |
|  | A911_07240 (Cj1502c) | -1.73 | 2.01E-71 | 1.05 | 0.60891718 |
|  | A911_07400 (Cj1537c) | -1.15 | 0.160056 | -1.29 | 4.74E-03 |
|  | A911_08320 (flgE) | 1.92 | 1.37E-09 | 2.27 | 0 |
|  | A911_08320 (flgE2) | 1.92 | 1.37E-09 | 2.27 | 0 |
|  | A911_00925 (Cj0191c) | -1.01 | 0.971014 | 1.24 | 2.98E-02 |
|  | A911_01240 (Cj0257) | 6.38 | 0 | 3.06 | 0 |
|  | A911_01835 (Cj0378c) | 2.94 | 0 | 1.85 | 1.77E-10 |
|  | A911_02485 (Cj0509c) | -2.48 | 1.34E-12 | -1.32 | 2.33E-02 |
|  | A911_02515 (Cj0515) | 2.28 | 0 | 2.26 | 0 |
|  | A911_03665 (hrcA) | -6.11 | 5.87E-31 | -3.28 | 5.43E-21 |
|  | A911_03670 (grpE) | -5.06 | 9.09E-133 | -2.71 | 7.66E-46 |
|  | A911_03675 (dnaK) | -6.60 | 1.12E-41 | -3.16 | 5.18E-22 |
|  | A911_03680 (Cj0760) | -1.11 | 0.117739 | -1.36 | 1.73E-05 |
|  | A911_03685 (Cj0761) | -1.05 | 0.415363 | 1.57 | 0.127623807 |
|  | A911_04310 (Cj0892c) | -1.53 | 2.61E-19 | -1.28 | 2.17E-03 |
|  | A911_05830 (Cj1202) | 2.48 | 1.77E-15 | 1.13 | 0.439314128 |
|  | A911_05920 (groES) | -1.70 | 3.88E-17 | -1.66 | 3.07E-13 |
|  | A911_05925 (groEL) | -1.98 | 0 | -1.90 | 0 |
|  | A911_05965 (cbpA) | 1.02 | 0.837946 | -1.60 | 1.80E-09 |
|  | A911_05970 (hspR) | -1.71 | 2.17E-18 | -1.94 | 8.70E-25 |
|  | A911_06290 (Cj1295) | 1.60 | 5.26E-15 | -1.14 | 7.68E-02 |
| NssR | A911_01510 (Cj0313) | -1.10 | 0.154811 | 1.12 | 0.124536997 |
|  | A911_02095 (Cj0430) | 1.26 | 8.28E-03 | 1.28 | 4.67E-03 |
|  | A911_02270 (ctb) | 1.15 | 9.24E-03 | 2.11 | 6.07E-02 |
|  | A911_03685 (Cj0761) | -1.05 | 0.415363 | 1.57 | 0.127623807 |
|  | A911_04025 (Cj0830) | 1.45 | 1.45E-03 | 1.25 | 0.095603249 |
|  | A911_04130 (Cj0851c) | 1.11 | 0.636902 | -1.00 | 0.995710887 |
|  | A911_06410 (Cj1319 ) | -1.69 | 2.52E-27 | -1.31 | 2.97E-06 |
|  | A911_06435 (Cj1324 ) | 1.20 | 4.62E-02 | -1.02 | 0.794000331 |
|  | A911_06440 (ptmC) | 2.11 | 0 | 1.31 | 9.43E-06 |
|  | A911_06470 (ptmB) | 3.30 | 0 | 1.37 | 1.60E-05 |
|  | A911_06480 (pseD) | 3.55 | 0 | 1.55 | 8.01E-04 |
|  | A911_06490 (P (maf4)) | 3.59 | 0 | 1.29 | 4.22E-02 |
|  | A911_07610 (Cj1582c) | -2.22 | 1.38E-03 | -1.13 | 0.590402516 |
|  | A911_07630 (cgb) | 1.68 | 4.59E-06 | 15.09 | 9.45E-02 |
| PerR | A911_00345 (Cj0073c) | -2.83 | 9.78E-29 | -1.36 | 2.07E-05 |
|  | A911_00430 (Cj0091) | 1.13 | 9.46E-02 | 1.65 | 0 |
|  | A911_00990 (Cj0204) | 1.44 | 6.45E-14 | 1.24 | 1.06E-02 |
|  | A911_01370 (cheA) | -2.87 | 0 | -1.61 | 2.35E-48 |
|  | A911_01555 (perR) | -1.55 | 3.36E-36 | -1.30 | 4.28E-08 |
|  | A911_01585 (fabH) | 2.04 | 0 | 1.70 | 3.04E-14 |
|  | A911_01590 (plsX) | 1.53 | 1.47E-08 | 1.49 | 2.40E-06 |
|  | A911_02130 (sdhA) | -2.61 | 5.36E-36 | -1.57 | 7.71E-02 |
|  | A911_02160 (accA) | 1.01 | 0.940487 | 1.32 | 1.14E-08 |
|  | A911_02610 (oorA) | -1.35 | 1.08E-07 | -1.25 | 2.58E-04 |
|  | A911_02615 (oorB) | -1.38 | 9.16E-10 | -1.20 | 7.35E-03 |
|  | A911_02915 (fba) | 1.72 | 0 | 1.55 | 0 |
|  | A911_02985 (Cj0611c) | -1.26 | 1.55E-03 | 1.09 | 0.237888065 |
|  | A911_03420 (Cj0701) | -1.33 | 1.08E-10 | -1.08 | 3.83E-02 |
|  | A911_03530 (Cj0723c) | 1.48 | 6.87E-05 | 1.77 | 0 |
|  | A911_03780 (napA) | -2.52 | 2.08E-249 | -1.75 | 1.53E-195 |
|  | A911_03785 (napG) | -2.69 | 4.19E-189 | -1.71 | 3.79E-163 |
|  | A911_03790 (napH) | -2.54 | 5.66E-32 | -1.49 | 7.19E-08 |
|  | A911_04045 (Cj0834c) | -4.52 | 3.74E-148 | -1.81 | 2.49E-45 |
|  | A911_04125 (Cj0850c) | 1.15 | 7.49E-02 | -1.14 | 0.195142767 |
|  | A911_05100 (murC) | -1.62 | 2.35E-11 | -1.91 | 1.24E-15 |
|  | A911_05915 (Cj0403) | 2.19 | 0 | 1.54 | 2.02E-12 |
|  | A911_05950 (Cj1226c) | 2.58 | 0 | 1.98 | 0 |
|  | A911_06085 (Cj1252) | 1.03 | 0.59661 | -1.62 | 4.19E-29 |
|  | A911_06435 (Cj1324) | 1.20 | 4.62E-02 | -1.02 | 0.794000331 |
|  | A911_06511 (Cj1341c) | 2.44 | 7.81E-12 | 2.11 | 6.11E-08 |
|  | A911_06525 (Cj1345c) | 1.11 | 0.237471 | -1.19 | 7.42E-02 |
|  | A911_06540 (Cj1342c) | 1.13 | 3.52E-02 | -1.22 | 3.06E-03 |
|  | A911_06590 (Cj1356c) | -2.95 | 3.99E-76 | -1.54 | 1.12E-03 |
|  | A911_07530 (nuoN) | -1.39 | 1.32E-03 | -1.14 | 0.446417718 |
|  | A911_00050 (Cj0011c) | -1.68 | 2.11E-05 | -1.14 | 0.29504788 |
|  | A911_00055 (Cj0012c) | -2.98 | 1.52E-19 | -1.37 | 1.65E-02 |
|  | A911_00195 (Cj0040) | 1.74 | 2.25E-12 | 2.19 | 1.60E-02 |
|  | A911_00200 (Cj0041) | 2.40 | 3.02E-14 | 2.72 | 2.90E-04 |
|  | A911_00205 (FlgD) | 2.01 | 6.21E-13 | 2.59 | 1.03E-03 |
|  | A911_00210 (FlgE) | 1.97 | 3.25E-07 | 2.10 | 1.65E-09 |
|  | A911_00215 (Cj0044c) | 2.90 | 0 | -3.89 | 2.44E-24 |
|  | A911_00220 (Cj0045c) | 2.62 | 1.01E-13 | -4.52 | 5.84E-12 |
|  | A911_00705 (trxB) | 1.23 | 1.65E-03 | -1.20 | 9.98E-04 |
|  | A911_00720 (Cj0148c) | -1.02 | 0.654601 | 1.11 | 3.34E-02 |
|  | A911_00840 (Cj0174c) | 5.25 | 0 | 3.83 | 1.44E-07 |
|  | A911_00845 (Cj0175c) | 4.30 | 0 | 2.96 | 1.07E-06 |
|  | A911_00855 (Cj0177) | 1.01 | 0.988839 | -1.32 | 0.388994463 |
|  | A911_00865 (exbB1) | 1.17 | 0.623487 | -1.35 | 0.417065545 |
|  | A911_00870 (exbD1) | 2.87 | 5.05E-03 | 1.27 | 0.652295601 |
|  | A911_01220 (Cj0253) | -1.09 | 0.420433 | -1.07 | 0.55619385 |
|  | A911_01595 (rpmF) | 1.04 | 0.643199 | 1.02 | 0.835017258 |
|  | A911_01615 (ahpC) | -2.09 | 1.30E-219 | -1.78 | 1.21E-121 |
|  | A911_02025 (Cj0416) | 1.62 | 3.33E-03 | 1.45 | 0.073540056 |
|  | A911_02085 (Cj0428) | -1.07 | 0.348389 | 1.34 | 8.38E-07 |
|  | A911_02415 (Cj0494) | 2.60 | 4.23E-10 | 2.50 | 0 |
|  | A911_02565 (flgC) | -1.03 | 0.407954 | -1.02 | 0.832824465 |
|  | A911_02570 (flgB) | 1.49 | 9.11E-05 | 1.79 | 4.58E-03 |
|  | A911_02700 (Cj0554) | -7.37 | 1.29E-25 | -2.81 | 3.95E-14 |
|  | A911_02855 (folP) | 1.76 | 0 | 1.26 | 2.52E-03 |
|  | A911_02995 (pstS) | 1.17 | 0.348545 | -1.25 | 0.250830582 |
|  | A911_03000 (pstC) | 1.24 | 0.343113 | -1.23 | 0.42501807 |
|  | A911_03345 (flgH) | 1.75 | 6.42E-04 | 2.06 | 0.144791124 |
|  | A911_03400 (flgG2) | 1.35 | 8.16E-03 | 1.64 | 0.180539551 |
|  | A911_03405 (flgG) | 2.10 | 0 | 1.51 | 1.25E-02 |
|  | A911_03450 (kdtA) | 1.45 | 0.012078 | 1.67 | 8.49E-10 |
|  | A911_03655 (tonB3) | 2.81 | 6.38E-04 | 1.14 | 0.564016549 |
|  | A911_03660 (cfrA) | -1.01 | 0.986476 | -1.17 | 0.48244142 |
|  | A911_03670 (grpE) | -5.06 | 9.09E-133 | -2.71 | 7.66E-46 |
|  | A911_03730 (Cj0770c) | -2.78 | 5.09E-63 | -2.11 | 3.39E-59 |
|  | A911_03950 (Cj0814) | 1.88 | 1.21E-06 | 2.02 | 1.29E-07 |
|  | A911_03965 (Cj0818) | 1.48 | 8.17E-05 | 1.61 | 1.37E-05 |
|  | A911_03975 (fliP) | -1.06 | 0.384501 | -1.08 | 0.255067089 |
|  | A911_04240 (Cj0877c) | -1.06 | 0.582068 | -1.11 | 0.13868563 |
|  | A911_04285 (flaD) | 2.01 | 6.14E-10 | 1.42 | 0.158689108 |
|  | A911_04350 (Cj0900c) | 1.17 | 0.13163 | 1.33 | 8.72E-04 |
|  | A911_04715 (Cj0977) | 2.19 | 0 | -1.75 | 2.71E-31 |
|  | A911_04820 (Cj0999c) | 1.16 | 0.252801 | 1.14 | 0.332115425 |
|  | A911_04940 (asd) | -1.61 | 4.98E-09 | -1.44 | 2.58E-06 |
|  | A911_04945 (Cj1024c) | -1.15 | 3.22E-03 | -1.26 | 2.31E-06 |
|  | A911_06280 (Cj1293) | 1.32 | 2.35E-08 | 1.31 | 0.177242294 |
|  | A911_06285 (Cj1294) | 1.44 | 4.21E-07 | 1.26 | 0.1758514 |
|  | A911_06290 (Cj1295) | 1.60 | 5.26E-15 | -1.14 | 7.68E-02 |
|  | A911_06570 (ceuC) | 2.20 | 1.70E-03 | 1.73 | 0.191647432 |
|  | A911_06720 (Cj1383c) | 1.31 | 0.537577 | 1.00 | 1 |
|  | A911_06725 (Cj1384c) | 1.36 | 0.450304 | -1.32 | 0.583405185 |
|  | A911_06730 (katA) | -3.05 | 3.35E-11 | -3.52 | 1.14E-16 |
|  | A911_07045 (flgI) | 1.34 | 8.09E-05 | 1.83 | 3.58E-02 |
|  | A911_07055 (Cj1464) | 1.36 | 4.93E-03 | -1.72 | 2.03E-08 |
|  | A911_07065 (flgK) | 2.12 | 1.13E-07 | 1.71 | 4.73E-09 |
|  | A911_07385 (Cj1534c) | -1.44 | 2.23E-38 | -1.02 | 0.51944493 |
|  | A911_07400 (acs) | -1.15 | 0.160056 | -1.29 | 4.74E-03 |
|  | A911_07440 (Cj1545c) | 1.18 | 3.85E-04 | -1.21 | 4.47E-04 |
|  | A911_07765 (Cj1613c) | 1.09 | 0.17776 | 1.06 | 0.381481522 |
|  | A911_07770 (chuA) | 1.11 | 0.776314 | -1.39 | 0.432589966 |
|  | A911_07775 (chuB) | 2.00 | 6.05E-05 | 1.58 | 1.69E-02 |
|  | A911_07780 (chuC) | 2.56 | 2.13E-02 | 1.18 | 0.777000406 |
|  | A911_07785 (chuD) | 2.76 | 5.24E-14 | 1.69 | 1.06E-03 |
|  | A911_07840 (exbB2) | 4.38 | 3.36E-04 | 1.47 | 0.395358842 |
|  | A911_07845 (exbD2) | 5.45 | 4.57E-07 | 1.56 | 0.288614614 |
|  | A911_07870 (aroC) | 2.91 | 0 | 1.61 | 2.03E-12 |
|  | A911_07990 (Cj1658c) | 6.12 | 0 | 2.49 | 0.128852991 |
|  | A911_07995 (p19) | 5.17 | 0 | 4.15 | 2.19E-02 |
|  | A911_08005 (Cj1661) | 10.44 | 0 | 7.72 | 2.42E-02 |
|  | A911_08015 (Cj1663) | 5.66 | 3.97E-13 | 3.84 | 1.19E-02 |
|  | A911_08020 (Cj1664) | 5.52 | 6.43E-10 | 3.91 | 1.42E-03 |

^*^Values in red are not significant
