# Supplementary material for: Host adaption to the bacteriophage carrier state of Campylobacter jejuni
Source: Res Microbiol. 2015 Jul-Aug;166(6):504–15. doi: 10.1016/j.resmic.2015.05.003 (PMC4534711; doi:10.1016/j.resmic.2015.05.003)
Supplement: Table S4 — crRNA sequences of self-derived spacers expressed from PT14CP8CS or PT14CP30AC. [file mmc5.docx]

**Table S3 crRNA sequences of self-derived spacers expressed from PT14CP8CS or PT14CP30ACS**

| **crRNA sequence** | **Target strand^1^** | **Match^2^** | **Gene product** | **Locus tag** |
| --- | --- | --- | --- | --- |
| **Native *C. jejuni* PT14 crRNAs 1-3** |  |  |  |  |
| AUAAUUUCUAAUUUCAUUUAUAACCUUUCA | + | (18/30) PT14 | Peptidoglycan-associated lipoprotein Omp18 | A911_00540 |
| UAGUAGCUAAGAAUAAAAUAAGAAACACUG | + | (16/30) PT14 | Apolipoprotein N acyltransferase | A911_05300 |
| UAGUAGCUAAGAAUAAAAUAAGAAACACUG | + | (15/30) PT14 | *ileS* - isoleucyl tRNA synthetase | A911_05135 |
| **CS8 acquired crRNAs** |  |  |  |  |
| UAAAAAUUUAAGCCCGCAAAGUCAAAUUUC | - | (30/30) PT14 | Hemin binding protein | A911_07785 |
| UGCUUAAAUCCCCAAGUUUUUCUAAAAAUU | + | (30/30) PT14 | Conserved membrane protein | A911_02540 |
| UAAAAUCUUUAAAAUAUUCUAAAUUUUUUU | + | (30/30) PT14 | PseE motility associated protein | A911_06495 |
|  |  |  |  |  |
| **CS30 acquired crRNAs** |  |  |  |  |
| UGGCUUCAUAUUUGAUAUAAGUACCACGAU | + | (30/30) PT14 | Putative tungsten ABC-transport system | A911_07415 |
| GGCAUUAUUGAGCUGGUGUUUGCUCUUUUG | + | (30/30) PT14 | Conserved hypothetical protein | A911_00810 |
| UGGCUCUAAAACUCCGCUCAUAUAAACCAA | + | (29/30) PT14 | Anaerobic C4-dicarboxylate transporter | A911_00415 |
| AAGGGGUUGGCUUUUUGGAUUCUUUUUUAA | + | (26/30) PT14 | nusG Transcription anti-terminator | A911_02310 |
|  |  |  |  |  |

1. Target strand indicates whether the spacer binds the coding (+) or non-coding (-) DNA strands of PT14.
2. Base pairing to PT14 chromosomal locations
